# Identification of two divergent swine Noroviruses detected at the slaughterhouse in North East Italy

**DOI:** 10.1186/s40813-020-00147-1

**Published:** 2020-04-16

**Authors:** Andrea Laconi, Lara Cavicchio, Luca Tassoni, Giovanni Cunial, Adelaide Milani, Martina Ustulin, Guido Di Martino, Mario Forzan, Mery Campalto, Isabella Monne, Maria Serena Beato

**Affiliations:** 1 Research and Development Laboratory, Istituto Zooprofilattico Sperimentale Delle Venezie, Legnaro, Padua, Italy; 2 Department of Comparative Biomedicine and Food Science, University of Padua, Legnaro, Padua, Italy; 3 Diagnostic Virology Laboratory, Department of Animal Health, Istituto Zooprofilattico Sperimentale Delle Venezie, Legnaro, Padua, Italy; 4 Epidemiology Department, Istituto Zooprofilattico Sperimentale Delle Venezie, Legnaro, Padua, Italy; 5 Diagnostic Laboratory, Istituto Zooprofilattico Sperimentale Delle Venezie, Via Bassa del Cuc 4, 33084, Cordenons, Pordenone, Italy; 6 Department of Veterinary Science, University of Pisa, Viale delle Piagge 2, 56124 Pisa, Italy

**Keywords:** Norovirus, Swine, Survey, Recombination, Slaughterhouse

## Abstract

Norovirus (NoV) has emerged as one of the major causative agents of non-bacterial, food- and water-borne gastroenteritis in humans, with the main genogroup involved in human outbreaks (GII), which has been detected worldwide in different animal species including swine. A four-month investigation at the slaughterhouse aiming to examine the presence of NoV in the swine in North-Eastern Italy, enabled the detection of two divergent Noroviruses (NoVs) (GII.P11) in two swine farms. This represents the first study in the swine population of North-Eastern Italy, which has paved the way for future integrated virological and epidemiological investigations on swine NoVs.

## Background

Noroviruses (NoVs) are a leading pathogen of food-borne diseases, which cause an estimated 23 million cases of illness annually in the USA, and > 90% of non-bacterial epidemic gastroenteritis cases worldwide [1, 2]. NoVs are transmitted through the ingestion of contaminated food or water, either via the faecal-oral route or via air-borne particles and contact with contaminated surfaces [3]. NoVs belonging to the *Caliciviridae* family are classified into ten genogroups (GI-GX), based on the variation of the major capsid protein (VP1), and are further divided into 49 genotypes [4]. The genetic diversity of the RNA-dependent-RNA-polymerase (RdRp) dictates the number of p-types [4]. NoVs detected in naturally infected pigs are classified as GII, which can also infect humans [5–9]. Since the first report of GII NoVs in pigs in the USA [10], other countries have reported the presence of this genogroup in diseased and healthy pigs [11]. However, little is known about the GII distribution and the epidemiological characteristics of NoVs in swine. Currently, no data are available on the NoVs prevalence in swine in Italy, although, belonging to the family *Caliciviridae*, they are listed among zoonotic agents that European member states should report on [12–15]. The aim of this study was to investigate the presence of NoVs in swine in two North East Italian regions, Veneto and Friuli Venezia Giulia (FVG).

## Materials and methods

Seventy-nine faecal samples from 76 swineherds were collected at three slaughterhouses between March and June 2017. Forty swine herds were distributed in Veneto (eighteen fattening, six farrow to wean and sixteen farrow to finish) and 36 in FVG (thirty fattening farms, one farrow to wean and five farrow to finish).

Faecal samples were diluted 1:5 (weight (w)/volume (v)) in Phosphate Buffered Saline (PBS) supplemented with antibiotics (10,000 IU/ml of penicillin G, 10 mg/ml of streptomycin, 5000 IU/ml nystatin, 0.25 mg/ml gentamicin sulphate) (Sigma Aldrich, St. Louis, Missouri, USA). The homogenates were diluted in a final 20% glycerol solution (v/v) (Sigma Aldrich, St. Louis, Missouri, USA), vortexed and centrifuged, and the supernatants collected, aliquoted and stored at − 80 °C. Viral RNA was isolated using QIAamp Viral RNA mini kit (QIAGEN, Hilden, Germany).

One-step RT-PCRs were performed using the SuperScript III One-Step RT-PCR System with Platinum Taq DNA polymerase kit (Invitrogen, Carlsbad, CA, USA). For NoV detection an approach based on a universal one-step RT-PCR for *Caliciviridae* family [10, 16] targeting a 300 bp RdRp fragment, paired with Sanger sequencing and BLAST search was adopted. Primers pair NVG4F and VN3T20 [10] were used to amplify a genomic portion of about 2500 bp containing the VP1 coding region, followed by sanger sequencing and BLAST search. A primer-walking approach based on newly designed primers (Table 1) was used to amplify and sequence NoV full genome applying the thermal cycling profile reported in Table 2.

**Table 1:** Primers used for reverse transcription, PCR and sequencing. Primer names generally indicate approximate binding positions in the NoVs genome

Primer	Sequence (5′-3′)	Genome Position (nt)	Amplicon size
NORP7F	GAAGATGGCGTCTAACGACG	7–27	539 bp
NORP_546R	TGAGGGACATGCACCACTC	527–546	
NORP_374F	GAACCACTCCCAGGCTCTAT	374–394	876 bp
NORP_1250R	CCTCCATGTCTAGAACAGCA	1230–1250	
NORP1085F	CGCATGTTCACTTCAGCAGC	1085–1105	951 bp
NORP2036R	CCCTTYCCATAAGGGGTGTT	2016–2036	
NORP3127F	CTTTTGATYACCACCACTCATGT	3127–3150	1191 bp
NORP4318R	TCTGTTGGGTGGAGTCCCA	4299–4318	
NORP3819F	GAAACCATAGTGAATTTTCTAG	3819–3841	540 bp
NORP4359R	CAGCAGAGAATTTCACCATG	4339–4359	
NORP4051F	TGAARGATGARCTTGTSAAGAC	4051–4073	1087 bp
NORP5138R	TTGACCTCTGGTACGAGACC	5118–5138	
NVG4F	TGGATGCGRTTCTCNGACYT	5007–5027	1821 bp
NORP5647F	CGAACAATGCTGGGGATGATGTTT	5647–5671	
NORP6828R	TGATTAAKKGCATTRGYACCAGCA	6804–6828	
NORP6579F	CACCACAGGTAGAGTGCTC	6579–6598	1071 bp
NORP7650R	TTTTTTTGGAGATCAGGGAACAG	7627–7650

**Table 2:** Thermal cycling profile used for one-step RT-PCRs developed in the present study as part of the *in-house* primer-walking approach to amplify the whole genome sequence of swine NoVs

Thermal cycling profile	Temperature	Duration	N° cycles
Reverse transcription reaction	50 °C	60 min	
Polymerase Activation	94 °C	2 min	
Denaturation	94 °C	15 s	X40
Annealing	50~60 °C	30 s	
Elongation	68 °C	1~2 min	
Final elongation	68 °C	5 min	

Nucleotide (nt) sequences of the partial RdRp and of the VP1 were aligned with the MAFFT online software version 7 (https://mafft.cbrc.jp/alignment/software/). The best substitution model was identified using MEGA 6.0 and the phylogenetic analysis was performed with PhyML 3.0 with 100 replicate of bootstrap.

The presence of recombination events was investigated using RDP4 software (http://web.cbio.uct.ac.za/~darren/rdp.html) using eight methods (RDP, GENECONV, BOOTSCAN, MAXCHI, CHIMAERA, SISCAN, PHYLPRO and 3SEQ) and ten representative NoV full genome sequences (JX023285.1 GI.1; X81879.1 GII.2; AB039781.1 GII.3; AB220921.1 GII.4; AY502023.1 GII.4; AB039779.1 GII.6; HQ169542.2 GII.6; HQ392821.1 GII.11; AY772730.1 GII.16; AY823305.2 GII.18).

## Results and discussion

**Fig. 1:**
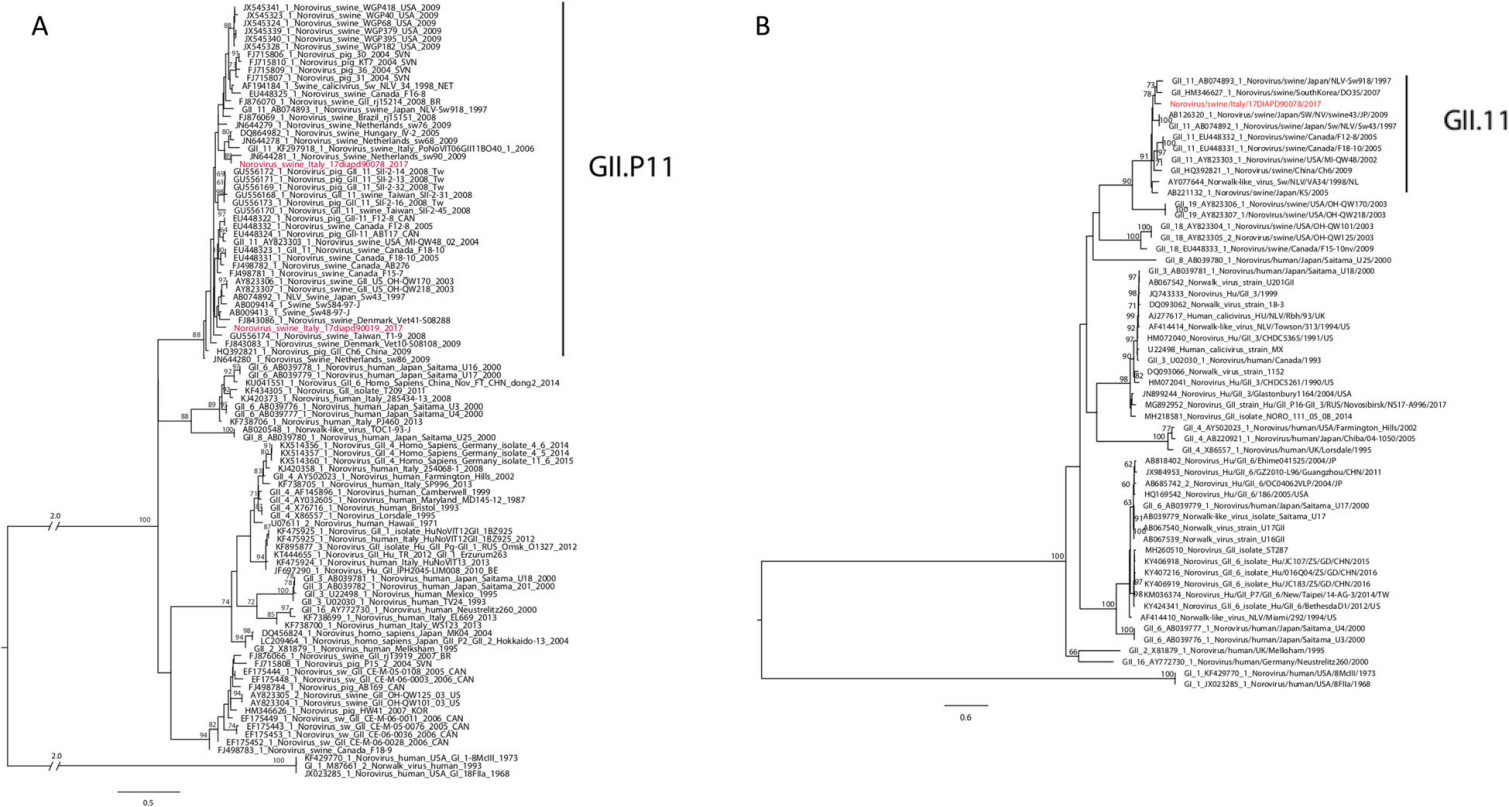
**a** Phylogenetic tree of the RdRp nucleotide sequence on the left. Nucleotide substitution model: GTR + G; 100 bootstrap replicate. Only bootstrap values equal or higher than 60 are showed. In red the viruses identified in the present study. **b** Phylogenetic tree of the VP1 nucleotide sequence on the right. Nucleotide substitution model: GTR + G + I; 100 bootstrap replicate. Only bootstrap values equal or higher than 60 are showed. In red the virus identified in the present study

Two out of seventy-nine faecal samples (2.53%) resulted NoV positive and characterized as GII.P11 based on the RdRp sequence (Fig. 1). NoVs were identified in two different fattening farms located in Veneto provinces in March and June 2017, namely: NoV/Italy/swine/17DIAPD90019/2017 (90019/2017) from Padova province and NoV/Italy/swine/17DIAPD90078/2017 (90078/2017) from Verona province. No NoV positive faecal samples were detected in FVG region.

The phylogenetic analysis based on 300 bp of the RdRp highlighted that the two Italian NoVs belong to the GII.P11 p-type but cluster into two different GII.P11 subgroups (Fig. 1a). The genetic similarity between 90078/2017 (accession number: MN567957) and 90019/2017 (accession number: MN567956) was 85.31%. The 90078/2017 NoV showed the highest nt similarity (91.70%) with Norovirus/Swine/Netherlands/sw90/2009 (JN644281), while 90019/2017 strain showed the highest similarity (89.74%) with Nov/Swine/Sw48–97-J (AB009413), suggesting that the two Italian samples were different from each other and from any other GII.P11 NoVs. At amino acid (aa) level the RdRp sequence of 90078/2017 NoV showed the highest similarity (98.9%) with Nov/Swine/Sw48–97-J, while the RdRp sequence of the 90019/2017 NoV was identical to the RdRp of several NoVs.

Based on the phylogenetic analysis of the VP1 strain 90078/2017 belongs to the GII.11 genotype, as it clusters with NoVs identified in Japan in 1997 and in South Korea in 2007 (Fig. 1b). It was not possible to obtain the VP1 sequence of strain 90019/2017. The genetic distance computed on the nt sequence of VP1 showed that the sequence of strain 90078/2017 was significantly different from those available in Genbank, showing the highest nt similarity with GII.11 Norovirus/swine/Japan/NLV-Sw918/1997 (AB074893) (88.97%) and the aa highest similarity (97.06%) with GII.11 Sw/NLV/VA34/1998/NL (AY077644). The comparison between 90078/2017 and the other GII.11 VP1 sequences available allowed the identification of six unique aa mutations (T224I, I290V, S336A, F370Y, R393K, T474A, aa positions in respect to Norovirus/swine/China/Ch6/2009).

The nearly complete genome sequence was obtained only for 90078/2017 and it was 7045 nt in length, with 18 and 500 nt missing toward the 5′ and the 3′ ends respectively, and organized in three open reading frames (ORFs). ORF1 showed the conserved cleavage sites and aa motifs typical of NoVs, including GRPGIGKT (nucleoside-triphosphatase (NTPase)), EYDEY (viral protein genome-linked (VPg)), GDCG (protease), and DYSRWDST, GLPSG and YGDD (RdRp). In accordance with the genome organization of other NoVs, ORF1 and ORF2 showed an overlap of twelve nt, with the latter encoding for the capsid protein in a + 1 frame.

**Fig. 2:**
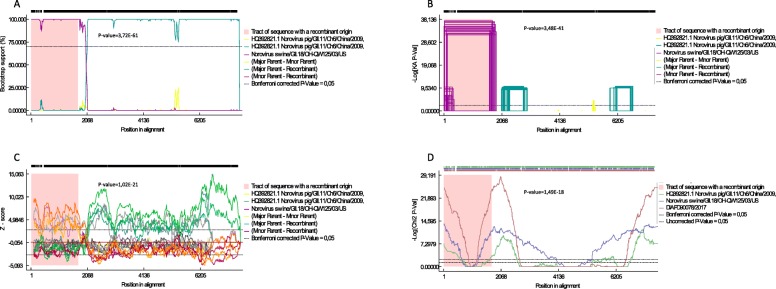
RDP4 plots obtained with (**a**) BOOTSCAN, (**b**) GENECONV, (**c**) SISCAN and (**d**) CHIMAERA methods. The left and right bounds of the pink region indicate breakpoints suggested by the method. For easiness of representation, only four out of eight RPD4 plots are reported

Recombination analysis showed the presence of a possible recombination event in the ORF1 with GII.P18 strain NoV/swine/GII.18/OH-QW125/03/US (AY823305.2). The recombination event was supported (*p* < 0.05) by seven out of eight methods with PhylPro being the exception (Fig. 2); however, the presence of double peaks in the chromatograms in the area surrounding the potential break point (1847 (G/C), 1852 (C/A), 1858 (A/G), 1863 (T/C) and 1875 (T/C)), hinders its confident identification. Sequencing of the genomic region of interest was repeated using freshly isolated RNA and adopting different primer combinations. However, it was not possible to obtain any sequence deprived of double peaks around the putative break point.

Up to date, the only detection of NoV in swine reported in Italy resulted from a retrospective analysis on archived faecal samples collected in 2006, which did not disclose sample characteristics, source and geographic origin and produced merely a 300 bp of the RdRp swine NoV sequence [17]. A follow-up study attempting to identify NoVs in swine in Italy proved unsuccessful [18]. Our investigation adds new and updated information on swine NoVs in Italy, obtained through sampling at the slaughterhouses. Although slaughterhouse sampling does have its limits, as primarily older animals are surveyed, it also presents several advantages in terms of animals and farms that can be potentially sampled, time and resources necessary, appearing a cost-effective approach to investigate the presence of NoVs for which epidemiological background information are missing in Italy.

NoV positivity (2.53%) was in accordance with the previous reports on the detection of NoV in swine in Europe [19, 20]; however, higher positivity rates were observed in studies conducted in Asia (> 10%) and North America (> 20%) [21–23].

Based on the partial RdRp sequence, the Italian strains belong to the GII.P11 p-type, proving, however, to be genetically distinct from GII.P11 NoVs reported worldwide. The two strains, sampled only few months apart from animals originating from different provinces, showed a low RdRp genetic identity (85.31%) between each other. This is suggestive of a high genetic variability among GII.P11 NoVs circulating in the area. This evidence, combined with the previous findings, which report that other age and production categories can harbour NoVs [19, 20, 23, 24], suggests that modifying the sampling strategy may have a remarkable impact on the number and type of NoVs detected in swine in Italy, possibly elucidating the association between NoV detection, animal categories and farm typology.

We reported the first European swine NoV nearly full genome sequence, obtained using an *in-house* primer-walking strategy. Full genome analysis revealed the presence of a possible recombination event in the ORF1. This process normally occurs in the ORF1/ORF2 overlap region [25], although recombination events in other genomic regions have also been reported [26]. The putative minor parental strain was identified as a virus belonging to the GII.P18/GII.18 genotype. Recombination events between NoV genotypes had been observed and represent a major threat for human and veterinary health, as novel recombinants might have different antigenic properties compared to their parental strains [27, 28]. However, our findings are not conclusive, as it was not possible to identify the recombination break point. Co-infections of NoVs belonging to different genotypes have been observed [25], hence the authors cannot exclude to be in the presence of a co-infection, rather than of a recombinant virus. The detection of GII.P18 genomic sequences suggests the circulation of this p-type in the area, making future investigations worthwhile.

NoV is largely studied as a human virus, only recently receiving attention for its capability of infecting terrestrial animals. Studies on NoV in swine remain isolated efforts, meaning that available information is disconnected by spatial-temporal gaps. Enhanced surveillance is uttermost necessary to explain the evolution and ecology of swine NoVs.

## Data Availability

Data requests should be directed to the corresponding author.

## Abbreviations

Aa: Amino acid
FVG: Friuli Venezia Giulia
NoV: Norovirus
nt: Nucleotide
NTPase: Nucleoside-triphosphatase
ORF: Open reading frame
PBS: Phosphate Buffered Saline
RdRp: RNA-dependent-RNA-polymerase
v/v: Volume/volume
VP1: Major capsid protein
VPg: Viral protein genome-linked
w/v: Weight/volume
